# Incidence of sickness absence by type of employment contract: one year follow-up study in Spanish salaried workers

**DOI:** 10.1186/s13690-016-0152-x

**Published:** 2016-09-27

**Authors:** Elena Zaballa, José Miguel Martínez, Xavier Duran, Constança Albertí, David Gimeno Ruiz de Porras, Fernando G. Benavides

**Affiliations:** 1 Department of Experimental and Health Sciences, Center for Research in Occupational Health (CiSAL), Universitat Pompeu Fabra (UPF), Parc de Recerca Biomèdica de Barcelona, C/ Dr Aiguader 88-Primera planta, Despacho 171.02, 08003 Barcelona, Spain; 2 CIBER in Epidemiology and Public Health (CIBERESP), Barcelona, Spain; 3 Hospital del Mar Medical Research Institute (IMIM), Barcelona, Spain; 4 Departament de Salut, Generalitat de Catalunya, Institut Català d’Avaluacions Mèdiques i Sanitàries (ICAMS), Catalonia, Spain; 5 Department of Epidemiology, Human Genetics and Environmental Sciences, The University of Texas Health Science Center at Houston, School of Public Health, San Antonio Campus, San Antonio, TX USA

**Keywords:** Employment conditions, Occupational health, Sick leave, Social Security system

## Abstract

**Background:**

To examine the differences in the incidence of registered sickness absence by type of employment contract in a large representative sample of salaried workers in Spain in 2009.

**Method:**

A study of 653,264 salaried workers covered by the Social Security system who had 133,724 sickness absence episodes in 2009. Crude and adjusted rate ratios and their corresponding 95 % confidence intervals (CIs) were calculated with Poisson regression models.

**Results:**

The incidence rate per 100 workers-year of sickness absence for temporary workers (IR = 32.2) was slightly higher than that of permanent workers (IR = 28.9). This pattern was observed in both men (RR = 1.12; 95 % CI 1.10–1.14) and women (RR 1.11; 95 % CI 1.09–1.12). However, after adjusting for age, company size, and occupational category, the differences disappeared in men (_a_RR = 1.01; 95 % CI 0.99–1.02) and decreased in women (_a_RR = 1.06; 95 % CI 1.04–1.07).

**Conclusion:**

Our findings provide evidence on the independence of sickness absence benefits from the type of employment contract as well as on the nonexistence of incentives for taking sickness absence in workers with a permanent employment contract. In the context of increasing market flexibility, these results show a positive functioning of the Social Security system.

**Electronic supplementary material:**

The online version of this article (doi:10.1186/s13690-016-0152-x) contains supplementary material, which is available to authorized users.

## Background

Increased labour market flexibility, mainly reflected by an increasing the number of temporary employees, is offered as a solution for the financial and economic crisis, especially in Europe [1]. In Spain, the use of temporary work contracts has become a commonplace strategy for achieving labour market flexibility, with temporary jobs accounting for 25.3 % of the total jobs created in Spain, the second highest rate in the European Union [2].

In the current global market economy, flexibility cannot occur in a vacuum and a balanced combination of employment flexibility and security has been suggested to offer labour market advantages [3]. Providing workers with social benefits, such as guaranteed income and health care during a sickness absence episode, irrespectively of their type of employment contract, could be a potential mechanism for ensuring employment security in the context of employment flexibility.

To assess this hypothesis, in a 1-year follow-up study we examined the differences in the incidence of registered sickness absence between a large representative sample of permanent and temporary salaried workers in Spain.

## Methods

The study was based on a cohort of 653,264 salaried workers with 133,724 medically-certified sickness absence episodes who were actively registered in the Spanish National Social Security System between 1 January and 31 December 2009. Anonymized data were drawn from the Spanish Continuous Working Life Sample [4] and the Social Security sickness absence register.

In Spain, all sickness absence registered episodes must be physician-certified and include health care from the first day of absence. To be eligible for wage replacement, the worker must have contributed at least 180 days in the previous 5 years before the episode. While this wage replacement will generally start on day 4^th^ of the absence, collective bargaining agreements may exist that guaranty coverage by the employer for the first 3 days. In all cases, the employer bears the cost between the 4^th^ and the 16^th^ day, and the National Social Security Insurance covers the cost from the 17^th^ day onwards up to a maximum of 12 months. This period can be extended six additional months if the Social Security determines the worker is expected to recover and return to work [5].

We calculated the incidence rate for the first episode of sickness absence, and corresponding 95 % confidence intervals (CIs), for permanent and temporary salaried workers, using as the denominator (person-time at risk) the sum of days each worker was affiliated with the Social Security as either a temporary or permanent employee. Analyses were stratified by sex, and adjusted by age (15–25, 26–45, or 46–64 years), firm size (1–10, 11–49, or ≥50 workers), occupational category based on the social insurance contribution (skilled non-manual and manual, and unskilled non-manual and manual) and economic activity based on the Statistical Classification of Economic Activities in the European Community (Agriculture, fisheries and extractive industries, Manufacturing, Production and distribution of energy, Building, Commerce, Catering trade, transport and telecommunications, Financial intermediation, Real estate activities, Public administration, Education, health activities, community service and activities at home).

Poisson regression models with robust error variances, stratified by sex and adjusted for the other covariates, were used to calculate the rate ratios (RR) and corresponding 95 % CIs. Given all the covariates were found to modify the association between type of contract and sickness absence incidence, a final regression model was adjusted for all the covariates. We also computed the percentage reduction in RR as (unadjusted RR-adjusted RR)/(unadjusted RR-1). All analyses were performed with Stata 10.1©.

## Results

The sickness absence incidence rate per 100 workers-year (see Additional file 1: Table S1) was slightly higher in temporary (32.2; 95 % CI: 31.9–32.6) than in permanent workers (28.9; 95 % CI: 28.7–29.1). This pattern of higher incident rates in temporary versus permanent workers was observed for age, except among workers in the older age group (26.9 vs. 29.0); occupational categories, but manual workers exhibited similar incidence rate of sickness absence irrespective of contract type (29.4 vs. 30.2 in skilled workers; 35.0 vs. 34.2 in unskilled workers); and economic activity, excluding workers in financial intermediation (18.9 vs. 20.0) and education, health activities, community service and activities at home (36.1 vs 36.3).

The overall risk of sickness absence was about 12 % higher in temporary than in permanent workers (Table 1) for both men (RR = 1.12; 95 % CI: 1.10–1.14) and women (RR = 1.11; 95 % CI: 1.09–1.12). The difference between temporary and permanent workers in men diminished after adjusting for age (_a_RR = 1.07; 95 % CI: 1.05–1.09) and occupational category (_a_RR = 1.03; 95 % CI: 1.01–1.05). In women the difference was reduced after adjusting for firm size (_a_RR = 1.07; 95 % CI: 1.05–1.09) and economic activity (_a_RR = 1.07; 95 % CI: 1.05–1.08). When all covariates were considered simultaneously, the differences disappeared in men (_a_RR = 1.00; 95 % CI 0.98–1.02) and were attenuated by 55 % in women (_a_RR = 1.05; 95 % CI 1.03–1.06).

**Table 1:** Rate ratio (RR) of sickness absence for temporary versus permanent workers in the Continuous Working Life Sample. Spain, 2009

		Men			Women		
		RR	95 % CI	% change	RR	95 % CI	% change
Crude		1.12	1.10–1.14	–	1.11	1.09–1.12	–
Adjusted for							
	Age (as continuous)	1.07	1.05–1.09	−41.7	1.11	1.09–1.12	0.0
	Firm size	1.14	1.12–1.16	+16.7	1.07	1.05–1.09	–36.4
	Occupational category	1.03	1.01–1.05	−75.0	1.11	1.09–1.13	0.0
	Economic activity	1.12	1.10–1.14	0.0	1.07	1.05–1.08	−36.4
	Age, firm size, occupational category, economic activity	1.00	0.98–1.02	−100	1.05	1.03–1.06	−54.5

## Discussion

In 2009, the incidence rate of sickness absence in Spain was slightly higher in temporary workers compared to permanent workers. More than half of this difference in the case of women and all the difference in case of men were explained by differences in age, firm size, occupational category and economic activities between temporary and permanent workers. Thus, the incidence sickness absence, and the related social benefits, appeared to be independent of the type of employment contract during the period under investigation.

Our findings contrast with the limited available prior research reporting that temporary workers had a lower prevalence or incidence of sickness absence than permanent workers [6, 7]. These results were partially attributed to a higher perception of job insecurity in temporary workers than in permanent workers regarding how taking sickness absences may affect their chances of a contract being renewed [8, 9]. But methodological differences with prior research may also explain the contradictory findings. One of those previous studies was cross-sectional [7] and the other one, while longitudinal, was limited to workers in ten Finnish hospitals [6]. Also, in comparison with prior research, limited sample size as well as representativity of industrial sectors, our study examined a large nationally representative sample of workers from all economic activities. But, most importantly, the main difference from prior studies is that our study was based on incidence rate estimates with accurate time-at-risk for each worker rather than on self-reported or incomplete assessments of time-at-risk. The remaining differences in women between temporary and permanent workers after covariate adjustment could be attributed to the women’s “double burden” related to family responsibilities [10], which should be most frequently carried out by women with temporary contracts. Future research should elucidate this hypothesis.

Our results should be interpreted in the context of the second year of the financial crisis in Spain, and they could be explained because the level of job insecurity (e.g., fear of dismissal) may have increased in permanent workers to levels similar to those in temporary workers. Alternatively, and not mutually exclusive, the provision of social protection in terms of granting sickness absence benefits may have been strengthened as a matter of fact, although a prolonged economic crisis may change this pattern. Future data will help to test these hypotheses.

Several limitations may have influenced our findings. First, regarding sickness absence eligibility, all workers needed to have contributed to the Social Security system for a minimum of 180 days in the last 5 years prior to the sickness absence episode [5], which may limit the access to sickness absence benefits for temporary workers. This is, however, an unlikely explanation for our findings given we observed temporary workers having higher incidence of sickness absence than permanent workers. Second, when measuring incidence, there is a possible selection bias related to discarding the most severe cases at baseline. Although we do not have information about prior health status, the very large sample size and the relatively long follow-up (12 months) minimizes this bias [11], while guarantying the inclusion of sickness absences with all levels of severity. Strengths of our study are being a longitudinal study with an exact person-time estimation, and being based on a large nationally representative sample of the Spanish workforce with an exhaustive register of all physician-certified sickness absence episodes.

## Conclusion

Our findings offer preliminary evidence that the Social Security system provides both wage replacement and health care, irrespectively of the type of employment contract. Also, our findings suggest that permanent employment contracts are not an incentive for taking sickness absence. Therefore, in the context of an increasing flexible labour market based on temporary employment such as the Spanish market, these findings results show a positive functioning of the Social Security system. Future research with longer follow-up periods and in other countries would need to confirm our findings.

## Abbreviations

_a_RR, adjusted rate ratio; CI, confidence interval; RR, rate ratio

## Additional file

Additional file 1: Table S1. Incidence rates (IR) per 100 workers-year of sickness absence (SA) by socio-economic and occupational characteristics by type of employment contract. Continuous Working Life Sample, 2009. (DOCX 18 kb)
