# Supplementary material for: Incidence of sickness absence by type of employment contract: one year follow-up study in Spanish salaried workers
Source: Arch Public Health. 2016 Sep 27;74:40. doi: 10.1186/s13690-016-0152-x (PMC5037859; doi:10.1186/s13690-016-0152-x)
Supplement: Additional file 1: Table S1. — Incidence rates (IR) per 100 workers-year of sickness absence (SA) by socio-economic and occupational characteristics by type of employment contract. Continuous Working Life Sample, 2009. (DOCX 18 kb) [file 13690_2016_152_MOESM1_ESM.docx]

| **Additional table**. Incidence rates (IR) per 100 workers-year of sickness absence (SA) by socio-economic and occupational characteristics by type of employment contract. Continuous Working Life Sample, 2009. | | | | | | | | | | |
| --- | --- | --- | --- | --- | --- | --- | --- | --- | --- | --- |
|  |  |  |  |  |  |  |  |  |  |  |
| **Characteristics** | **Temporary workers** | | | | | **Permanent workers** | | | | |
|  | **Workers-year** | **SA cases** | **%** | **IR** | **95%CI** | **Workers-year** | **SAcases** | **%** | **IR** | **95% CI** |
| **Gender** |  |  |  |  |  |  |  |  |  |  |
| Male | 66,564 | 17,829 | 45.8 | 26.8 | 26.4-27.2 | 185,609 | 44,486 | 46.9 | 24.0 | 23.7-24.2 |
| Female | 54,157 | 21,101 | 54.2 | 39.0 | 38.4-39.5 | 142,779 | 50,308 | 53.1 | 35.2 | 34.9-35.5 |
| **Age**(in years) |  |  |  |  |  |  |  |  |  |  |
| 16 - 25 | 24,771 | 9,531 | 24.4 | 38.5 | 37.7-39.3 | 25,963 | 9,238 | 9,8 | 35.6 | 34.9-36.3 |
| 26 - 45 | 71,383 | 22,802 | 58.6 | 31.9 | 31.5-32.4 | 201,939 | 56,427 | 59.5 | 27.9 | 27.7-28.2 |
| 46 - 64 | 24,567 | 6,597 | 17.0 | 26.9 | 26.2-27.5 | 100,486 | 29,129 | 30.7 | 29.0 | 28.7-29.3 |
| **Firm size** |  |  |  |  |  |  |  |  |  |  |
| 10 or less workers | 37,212 | 9,47 | 24.3 | 25.4 | 24.9-26.0 | 97,310 | 20,267 | 21.4 | 20.8 | 20.5-21.1 |
| 11- 49 workers | 27,230 | 8,066 | 20.7 | 29.6 | 29.0-30.3 | 77,763 | 20,615 | 21.8 | 26.5 | 26.1-26.9 |
| 50 or more | 56,279 | 21,394 | 55.0 | 38.0 | 37.5-38.5 | 153,315 | 53,912 | 56.9 | 35.2 | 34.9-35.5 |
| **Occupational category** |  |  |  |  |  |  |  |  |  |  |
| Skilled Non-manual | 21,388 | 5,784 | 14.8 | 27.0 | 26.4-27.7 | 73,700 | 16,855 | 17.8 | 22.9 | 22.5-23.2 |
| Skilled Manual | 41,017 | 12,048 | 31.0 | 29.4 | 28.9-29.9 | 97,076 | 29,278 | 30.9 | 30.2 | 29.8-30.5 |
| Unskilled Non-manual | 32,447 | 12,05 | 31.0 | 37.1 | 36.5-37.8 | 124,908 | 37,464 | 39.5 | 30.0 | 29.7-30.3 |
| Unskilled Manual | 25,869 | 9,048 | 23.2 | 35.0 | 34.3-35.7 | 32,704 | 11,197 | 11.8 | 34.2 | 33.6-34.9 |
| **Economic Activity** |  |  |  |  |  |  |  |  |  |  |
| Agriculture, fisheries and extractive industries | 934 | 217 | 0.6 | 23.2 | 20.2-26.5 | 2,033 | 385 | 0.4 | 18.9 | 17.1-20.9 |
| Manufacturing | 11,116 | 3,493 | 9.0 | 31.4 | 30.4-32.5 | 55,211 | 16,123 | 17.0 | 29.2 | 28.8-29.7 |
| Production and distribution of energy | 1,385 | 467 | 1.2 | 33.7 | 30.7-36.9 | 4,280 | 1,36 | 1.4 | 31.8 | 30.1-33.5 |
| Building | 22,106 | 5,525 | 14.2 | 25 | 24.3-25.7 | 23,266 | 5,379 | 5.7 | 23.1 | 22.5-23.7 |
| Commerce | 11,686 | 3,979 | 10.2 | 34.1 | 33.0-35.1 | 66,774 | 18,336 | 19.3 | 27.5 | 27.1-27.9 |
| Catering trade, transport and telecommunications | 15,532 | 4,894 | 12.6 | 31.5 | 30.6-32.4 | 50,717 | 13,778 | 14.5 | 27.2 | 26.7-27.6 |
| Financial intermediation | 774 | 146 | 0.4 | 18.9 | 15.9-22.2 | 13,024 | 2,604 | 2.8 | 20.0 | 9.2-20.8 |
| Real estate activities | 18,378 | 6,309 | 16.2 | 34.3 | 33.5-35.2 | 40,231 | 10,935 | 11.5 | 27.2 | 26.7-27.7 |
| Public administration | 12,067 | 4,250 | 10.9 | 35.2 | 34.2-36.3 | 22,791 | 7,715 | 8.1 | 33.9 | 33.1-34.6 |
| Education, health activities, community service and activities at home | 26,743 | 9,650 | 24.8 | 36.1 | 35.4-36.8 | 50,061 | 18,179 | 19.2 | 36.3 | 35.8-36.8 |
| **Total** | 120,721 | 38,930 | 100 | 32.2 | 31.9-32.6 | 328,388 | 94,794 | 100 | 28.9 | 28.7-29.1 |
